# Supplementary material for: The impact of climate change on the agriculture and the economy of Southern Gaul: New perspectives of agent-based modelling
Source: PLoS One. 2024 Mar 27;19(3):e0298895. doi: 10.1371/journal.pone.0298895 (PMC10971770; doi:10.1371/journal.pone.0298895)
Supplement: S1 Text — (DOCX) [file pone.0298895.s001.docx]

S1. ODD protocol for ROMCLIM

The model description follows the ODD (Overview, Design concepts, Details) protocol for describing individual- and agent-based models [1], as updated by Grimm et al. (2020) [2].

# Overview

## 1. Purpose and patterns

***The purpose of this model*** is to test the impact of the Roman Climate Optimum (RCO) (mid 3^rd^ c. BC-4^th^ c. AD) and then the climatic cooling of Late Antiquity (LALIA) (5^th^-7^th^ c. AD) on the rural economy of the Roman Empire. ROMCLIM simulates the potential impact of these climate changes on the commercial productions and incomes of vine, olive oil and cereals (the "Mediterranean triad"), which are considered as the main source of wealth in the Roman period in the Mediterranean.

We evaluate our model by its ability to reproduce three main **patterns**: **1**) climatic variations each year or century, **2**) variations of potential yields (for wine, olive oil and cereals) in function of these climatic change, **3**) production and profits made by agricultural holdings, mainly calculated according to variations of potential yields, prices of goods, costs of production and transportation.

## 2. Entities, state variable and scales

### 2.1. Description of the model’s entities and state variable

***Agents/individuals*** represent capitals of cities and three types of agricultural entities: wineries, olives groves and cereals farms. *Capitals of cities* created as agents represent area of consumption but do nothing themselves in the model. Their virtual existence just allows to agricultural exploitations to calculate distance between them. Main characteristics of *Wineries* and *Olive groves* are programmed according to a literal description given by Latin agronomist Cato (*De Re Rustica*, X-XI) in the 2^nd^ c. BC. *Farms* are familial exploitations producing cereals for their own consumption, keep some seed for sowing next year and sell the potential surpluses on the city market. The *state variables* of the three types of agents are their geographical location which defined the distance to nearest capital of city, their annual production, and the potential benefits they make every year from the selling of these productions (Table 1).

Each year the three kinds of agricultural holdings make a harvest and sell vine, olive oil and cereals in the nearest capital of city. For all the three types of exploitation, the annual harvest volume depends on the fluctuating yields linked to the climate change. In output of the model, the profit made by each type of exploitation is calculated by subtracting to the harvest market price the production cost and transport cost to the urban cities.

***Grid cells/spatial units*** represent a piece of square cultivated land of 8 x 8km (*i.e.* 64 km^2^) (but large part of them represent the Mediterranean to). They contain numerous paleoclimatic data (312 monthly temperatures and precipitation for each of the 13 centuries taken in account). Their *state variables* (Table 2) are potential yields values calculated by the model (for cereals, wine & olive oil) from paleoclimatic data, but also other data imported from several shapefiles (see § 6 ).

The ***observer*** (or ***global environment***) can modify during the simulations some parameters (seasonal temperatures and precipitation, market prices of agricultural goods, transport cost ratio,…) and observe the effects on the process of creation/abandonment of agricultural exploitations and on the fluctuations of their potential benefits.

### 2.2. Description of the model’s spatial and temporal scales.

***Spatial scales***. Each square cell of the model represents 8 km x 8 km in size (*i.e.* 64 km^2^). This resolution is kept from the original climate model ALADIN used in the model. The model’s spatial extent focusing on southern France is a rectangle of 66 x 34 square cells (*i.e.* 528 km x 272 km). Hence, the area represented is 143 616 km^2^. The simulation window is divided into two types of grid cells: Mediterranean water and land cells.

- *Water*: These cells take 715 cells (45 760 km^2^) to the southern end of the simulation window. Water cells currently are not used in the simulation and are kept for aesthetic purposes.
- *Land cells*: These cells take 1529 cells (97 856 km^2^) in the rest of the simulation window. These cells correspond to arable land and mainly contain climatic and potential yields values.

The model’s space is represented as bounded.

***Temporal scales***. The model runs at a 1-year time step, or at a 1-century time step (observer can switch between the two, depending on the purpose). The simulations run for a maximum of 13 centuries from the 6^th^ c. BC to the 7^th^ c. AD.

## 3. Process overview and scheduling

From the start to the end of the simulation, the different types of agricultural entities (wineries, olive groves and cereal farms) make each year a harvest which is sold (in whole or in part) on urban markets. All the entities acting at the same time and state variables are updated each year. Everything is synchronous. The volume of these harvests fluctuates annually based on potential yields and climate change. The model calculates (in sestertii) at each time-step the profits made by agricultural entity by subtracting to the market value of its harvest the different staff costs and the transport costs to the nearest urban market. The profits generated by an agricultural exploitation are represented on the simulation window by a square whose size is proportional to their value (larger is the square, higher is the potential profits). Exploitations remain in each cell as long as they manage to make a profit. If these benefits become negative, the farms disappear. However, they have the possibility of reappearing if yields become sufficient again at some point in the simulation. These disappearances or appearances lead to variations in the number of agricultural exploitations.

# Design concepts

## 4. Design concepts

***1. Basic principles****/research hypotheses.* The ROMCLIM model tests a set of hypotheses concerning the farming systems and agricultural geography of the southern Gaul countryside, as well as the potential impact of climate change on Roman agriculture and economy. Research on agricultural production in southern Gaul was particularly marked by the work of D. Garcia for the Iron Age and those of J.-P. Brun for the Roman period (see references in the main article). In the 1^st^ century AD, the Greek geographer Strabo described the landscape of Narbonne as like that of Italy by the development of vine and olive cultivation. Archaeological research undertaken in recent decades has confirmed the omnipresence of viticulture but has nevertheless led to question the importance of olive growing, now considered marginal. Within the "Mediterranean triad" (wine, olive tree, cereals), which formed the basis of the agricultural economy in southern Gaul (as elsewhere in the Mediterranean), the importance and place of cereal cultivation is still difficult to perceive by archaeology. The issue of cereal cultivation has long been problematic. It has been envisaged that this could have been practiced on the margins of large estates by farmers working on small family farms.

While agriculture is generally considered the main source of wealth for the Roman economy, its orientation preferentially subsistence or commercial remains problematic. For southern Gaul, the image of an "autarkic" rural economy practicing a polyculture of self-subsistence is still persistent in most of archaeological publications. However, it is now recognized that they could also practice specialized crops for trade, especially vines. The importance of commercial agriculture remains still difficult to assess: was it practiced in all rural estates? Was it geographically confined to a few areas very limited or was it spread homogeneously throughout *Gallia Narbonensis*? Finally, if the impact of these climate changes on cereal yields has been discussed [3], the question of their potential effects on the cultivation of vines and olive trees had never (to our knowledge) been raised before.

It should be recalled that in recent decades, paleoclimatology has highlighted significant climatic pulsations whose impact on societies is now being discussed. After the phase of strong global warming (mid-3^rd^ century BC - 4^th^ century AD) of the Roman Climate Optimum (RCO), the cooling of the Late Antique Little Ice Age (LALIA) reached its peak in Western Europe at the beginning of the Middle Ages (VI^th^-VII^th^ century AD).

ROMCLIM makes it possible to test the potential impact of these climate variations on agricultural yields, the profitability of different crops and the economy by mainly modelling variations in temperature and rainfall, as well as the functioning of Roman agricultural entities specialized in one type of production. For each cell of the model, these different entities can be considered as independent from each other or working together inside a villa estate as specialized annex.

*Literature/concept for agent behaviours.* The characteristics and functioning of the agents (*i.e.* agricultural entities) are taken from information provided to us by Latin historical sources. For the programming of wineries and olive groves, we have mainly based ourselves on the indications provided in the 2^nd^ century BC by Cato in his work *De Re Rustica* (Books X and XI). He gives a very detailed description of the equipment and personnel required for an olive grove of 14 people for 240 jugera (60 ha), as well as a vineyard of 100 jugera (25 ha) operated by 13 people. In these rentier exploitations, the annual cost of personnel is calculated on the basis of 140 sestertii per slave (according to Etienne 1980 [4]). For the modeling of cereal farms, we relied on the (unpublished) thesis of P. Ouzoulias (2006) [5], which describes in detail the theoretical functioning of a small ancient family farm.

***2. Emergence***. The model produces as emergent phenomena a virtual map of cultivated landscape or more precisely a map of economic potentiality for cultivation of vineyards, olives and cereals.

***3. Adaptation****.* Not implemented.

***4. Objectives.*** Not yet implemented in this first version.

***5. Learning***. Not implemented.

***6. Prediction.*** Not yet implemented in this first version.

***7. Sensing*.** Not implemented.

***8. Interaction***. Not yet implemented in this first version.

***9. Stochasticity***. The model is very few stochastic. We have only implemented an (optional) function that allows the precipitation and temperature values to fluctuate randomly annually until ± 20% around the seasonal averages.

***10. Collectives***. The model includes no collectives.

***11. Observations***. During the simulation, the observer can vary as he wishes from the interface the various modifiable parameters concerning the price of food, transport costs as well as temperatures and precipitation to empirically experiment the effect of these changes. Several graphs make it possible to control the values of these parameters (average yields, fluctuations in temperature and precipitation) and their effects on variations in the number of agricultural holdings and total potential income from viticulture, olive growing and cereals.

The map of areas of higher/lower profitability for the different types of crops obtained may have a predictive value for the location of agricultural holdings (if we admit that the landowners of Roman antiquity could be acted by a form of economic rationality). To optimize their income, they could have sought to acquire or set up specialized agricultural holdings in areas as close as possible from the market sites (to minimize transport costs) and the most favourable from a climatic and environmental point of view for the type of crop chosen (vine, olive or cereals).

The comparison of the geographic distribution of (bio)archaeological data (imported in the model, see § 6) attesting of different crops with the maps generated by the model can therefore allow us to determine whether the choice of the location of agricultural holdings made by landowners in antiquity consciously took into account (or not) a potential for profitability determined by the environment, climate and geography.

# Details

## 5. Initialization

- During the instantiation phase of the model, each terrestrial cell generates a winery, an olive grove and a cereal farm.
- Capitals of cities are generated in some cells according to a location map (imported by a vector layer, see § 6)
- The monthly temperature and precipitation values for each of the 13 centuries considered are imported from raster files and stored in cells/patches.
- The potential yields for vines, olive trees and cereals are directly calculated in each cell from temperature and precipitation data, thanks to an emulation function of the agro-ecosystem model Lund-Potsdam-Jena-managed-Land (LPJmL).

## 6. Input data

The input data of the model consists of 312 raster files (*.asc) of climatic data and 4 vector layers (*.shp):

***Climate data***. The imported raster files (*.asc) contain monthly averages of temperature and precipitation for each of the 13 centuries of the chronological sequence considered (6^th^ century BC – 7^th^ century AD). They were created from the crossing and processing under the R software (Raster package) of a low-resolution paleoclimatic reconstruction and a high-resolution modeling of the current climate (the ALADIN-climate model) (Fig 1).

- The paleoclimatic reconstruction used was carried out on a European scale using pollen data at a resolution of 5 minutes latitude and 5 minutes longitude (Guiot and Kaniewski 2015). We extracted from this large database the points located in southern France which we interpolated to create a raster file at the same resolution that the ALADIN-climate model.
- Developed by the National Center for Meteorological Research, the ALADIN-climate model, which covers the entire metropolitan France, is made up of 8602 points spaced 8 km apart in latitude and longitude. To create a reference model, we used monthly averages of rainy days (NORRR1MM) and temperature (NORTAV) between 1976 and 2005. The files have been downloaded from the following addresses:
- Temperatures: <https://www.data.gouv.fr/fr/datasets/r/10def999-5480-446f-a279-080df7b20a6a>
- Precipitation: <https://www.data.gouv.fr/fr/datasets/r/4384ed43-80a7-4d50-9d64-9a7262e8546f>

***Geographical and archaeological data***. The model also imports 4 vector layers (*.shp format) containing:

- The capitals of city of Roman Gaul (produced by P. Ouzoulias).
- Boundaries of the province of *Gallia Narbonensis* (Narbonnaise2.shp).
- Archaeological data related to viticulture (Attestation_viticulture.shp).
- Archaeological data related to olive oil production (sites_oleicoles_Var_Brun.shp).

## 7. Submodels

### LPJmL emulation:

We have coded under NetLogo a function (procedure) of emulation of the agro-ecosystem model LPJmL which makes it possible to calculate the potential yields of the vine, the olive tree and cereals in rainfed (non-irrigated) cultivation systems.

This function applies three equations (presented below) based on a regression of the actual outputs of the LPJmL model on seasonal averages of temperature (Thiv, Tprt, Tete, Taut) and precipitation (Phiv, Ppt, Pete, Paut) (Table 3) and calibrated on the 13 time slices between 2500 yr BP and 1300 yr BP (by steps of 100 years).

For each of the crops considered in the modelling, different coefficients were assigned to the seasonal averages of temperature and precipitation calculated by the model from monthly values. These monthly values imported into NetLogo come from files we created from a paleoclimatic reconstruction (see § 6).

- **Seasonal temperatures and precipitation**: the values of these parameters (Table 3) are calculated for each season (spring, summer, autumn, winter) and for each century by the average of monthly precipitation and temperature values imported into the model (see § 6).
- **Potential yields** for vines, olive trees and cereals are calculated at each time step and for each patch by equations integrating temperature and seasonal precipitation data:

The linear equations are the following (the squared R indicate the proportion of variance of the yield explained by the climate):

-Potential yields for rainfed cereals (T/ha) (R2=0.63):

$$PyCer=0.3452+0.033\times Thiv+0.0072 \times Tprt+0.0467 \times Tete-0.0523 \times Taut+0.0016 \times Pprt+0.0004 \times Paut$$

-Potential yields of rainfed vines (Hl/ha) (R2=0.75):

$$PyVine= -34.2138-2.64 \times Thiv+2.2907 \times Tprt-2.4294 \times Tete+5.3971 \times Taut-0.0033 \times Phiv+0.04 \times Pprt+0.0631 \times Pete-0.0184 \times Paut$$

-Potential yields for rainfed olive tree (Hl/Ha) (R2=0.94):

$$PyOli= -0.0553+0.053 \times Thiv-0.0776 \times Tprt-0.0099 \times Tete+0.0445 \times Taut-0.00004 \times Phiv+0.0016 \times Pprt+0.0026 \times Pete+ 0.0005 \times Paut$$

Figure 2 shows the comparison of the emulator and the simulations done by LPJmL on all the time slices. It shows the difficulty to reproduce with the emulator the extreme yields. In particular, it underestimates the high values of vines and olives and it overestimate the low values of wheat.

### For the three types of agricultural exploitation:

- **Harvest:** each type of agricultural holding carries out an annual harvest, the volume of which is calculated (in tons) by multiplying the area farmed (different for each type of exploitation) by the value of potential yields stored in the patch underlying the holding.
- **Market value of the harvest:** calculated (in sestertii) by multiplying the harvest by a chosen market price in a range which maximum is given by the edict of Diocletian (Table 4).
- **Distance to town:** distance between each type of agricultural exploitation and the nearest city capital is calculated by NetLogo.
- **Transportation costs:** are obtained by multiplying the annual production weight * distance to town * a cost/unity of weight/unity of distance. This last value is chosen by the observer in a range which the maximum is given by the edict of Diocletian: 20 denarii/mile/cart of 1200 pounds (which we have converted to 0.14 sestertii/kg/km).
- **Profits:** are calculated for each type of agricultural exploitation by subtracting transportation costs and staff costs (only here for wineries and olive groves) to the market value of production.
- **Disappearance/reappearance:** Grove wineries and olives disappear when profits turn negative due to a sharp drop in yields. Cereal farms disappear when it is no longer possible to feed the whole family. However, these types of exploitation may reappear in a cell when yields rise again above the threshold of profit or subsistence.

### For wineries & olive groves:

- **Staff costs [annual, static]:** are calculated (in sestertii) by multiplying the number of workers in each exploitation type (13 for wineries, 14 for olive groves) * an annual cost per person. For this last one, we have retained (as already mentioned) the value of 140 sestertii/person/year. So, the annual staff cost is static: 1820 sestertii/year for wineries and 1960 sestertii/year for olive groves.

### For cereal farms:

- **Annual consumption [1.2 tons]:** calculated by multiplying the number of people in each farm (n=6) * ratio of 200kg/year/person that we have defined according to the study of historical data.
- **Seeds [tons]:** the quantity of seeds keep each year by farmers for reseed fields the following year is calculated by multiplying the area cultivated * a ratio of 0.135 t/ha (which corresponds to the indications given by Columella (*De Re Rust*.,II, 9) to sow 5 modius of naked wheat/jugera).
- **Surplus [tons]:** are calculated by subtracting to the annual cereal harvest the quantity of seed and the annual consumption of the whole family.
