# Supplementary material for: The impact of climate change on the agriculture and the economy of Southern Gaul: New perspectives of agent-based modelling
Source: PLoS One. 2024 Mar 27;19(3):e0298895. doi: 10.1371/journal.pone.0298895 (PMC10971770; doi:10.1371/journal.pone.0298895)
Supplement: S2 Text — (DOCX) [file pone.0298895.s002.docx]

# S2. Sensitivity Analysis (SA) for ROMCLIM

**Method**

To prioritize the parameters influencing the outputs of agent-based models from the most to the least, sensitivity analyses are increasingly performed [7-10]. Among the different existing methods, we used for ROMCLIM the one-factor-at-a-time (OFAT) approach. We have thus varied in turn, and one by one, each of the main four parameters of the model (temperatures, precipitation, market price, transport costs) between its minimum value and its maximum value, keeping the others constant. This method has the advantage of being robust and relatively simple to implement, although it has the well-known disadvantage of not being able to highlight possible interactions between factors [8].

For climate, we pushed temperatures up to ± 2°C and precipitation up to ± 100 mm around the average values used in the model, which corresponds to an amplitude calculated from variations in annual paleoclimatic reconstructions. For vines, olive trees and cereals, we have varied prices and transport costs between a minimum equal to 0 and the maximum given for each of these agricultural commodities by the Edict of Diocletian (Table 1).

We present here the analysis carried out for only one of the thirteen centuries of the chronological sequence (here the third century AD), the results obtained being similar for all the others. Now frequently used for this type of analysis, these results are presented as Tornado diagrams produced under R with the ggtornado() function of the "Miscellaneous R Functions" package [11]. These diagrams are presented in the form of horizontal bars on either side of a baseline, which here corresponds to the results obtained with the average values of all parameters (Fig 1).

**Results**

The results of the sensitivity analysis are different for each of the three cultures (Fig 1). Viticulture is primarily sensitive to rainfall (P), while variations in market prices (MP) are preponderant for olive and cereal growing. Temperatures (T) – always less influential than rainfall – rank last for cereal cultivation and third for viticulture and olive growing. For the latter, transport costs (TP) occupy the last place, but come in second place for cereal cultivation, which is more sensitive to economic than climatic parameters.
