# Supplementary material for: The impact of climate change on the agriculture and the economy of Southern Gaul: New perspectives of agent-based modelling
Source: PLoS One. 2024 Mar 27;19(3):e0298895. doi: 10.1371/journal.pone.0298895 (PMC10971770; doi:10.1371/journal.pone.0298895)
Supplement: S3 Text — (DOCX) [file pone.0298895.s003.docx]

# S3. References for SI

[1]. Grimm V, Berger U, Bastiansen F, Eliassen S, Ginot V, Giske J, et al. A standard protocol for describing individual-based and agent-based models. Ecological Modelling. 2006;198(1-2): 115-126. doi: 10.1016/j.ecolmodel.2006.04.023.

[2]. Grimm V, Railsback SF, Vincenot CE, Berger U, Gallagher C, DeAngelis DL, et al. The ODD Protocol for Describing Agent-Based and Other Simulation Models: A Second Update to Improve Clarity, Replication, and Structural Realism. Journal of Artificial Societies and Social Simulation. 2020;23(2):7. doi: 10.18564/jasss.4259.

[3]. Harper K. The Fate of Rome: Climate, Disease, and the End of an Empire. Princeton: Princeton University Press; 2017.

[4]. Etienne R. La comptabilité de Columelle. Les « dévaluations » à Rome. Époque républicaine et impériale. Volume 2. Actes du Colloque de Gdansk (19-21 octobre 1978). Rome: Publications de l'École française de Rome; 1980. p. 121-28.

[5]. Ouzoulias P. L'économie agraire de la Gaule: aperçus historiographiques et perspectives archéologiques. Unpublished PHD, The University of Franche-Comté. 2006. Available from: <https://tel.archives-ouvertes.fr/tel-00011567/document>.

[6]. Guiot J, Kaniewski D. The Mediterranean Basin and Southern Europe in a warmer world: what can we learn from the past? Front. Earth Sci. 2015;18. doi: 10.3389/feart.2015.00028.

[7]. Brouwer Burg M, Peeters H, Lovis WA, editors. Uncertainty and Sensitivity Analysis in Archaeological Computational Modeling. Berlin: Springer-Verlag; 2016.

[8]. ten Broeke G, van Voorn G, Ligtenberg A. Which Sensitivity Analysis Method Should I Use for My Agent-Based Model? Journal of Artificial Societies and Social Simulation. 2016;19(1):5. doi: 10.18564/jasss.2857.

[9]. Niida A, Hasegawa T, Miyano S. Sensitivity analysis of agent-based simulation utilizing massively parallel computation and interactive data visualization. PLoS ONE. 2019;14(3): e0210678. doi.org/10.1371/journal.pone.0210678.

[10]. Kanters H, Brughmans T, Romanowska I. Sensitivity analysis in archaeological simulation: An application to the MERCURY model. Journal of Archaeological Science: Reports. 2021;38: 102974. doi:10.1016/j.jasrep.2021.102974.

[11]. Helveston JP. Miscellaneous R Functions. R package, version 1.5.0. 2021. Available from: <https://rdrr.io/github/jhelvy/jhelvyr/>
