# Supplementary material for: The impact of climate change on the agriculture and the economy of Southern Gaul: New perspectives of agent-based modelling
Source: PLoS One. 2024 Mar 27;19(3):e0298895. doi: 10.1371/journal.pone.0298895 (PMC10971770; doi:10.1371/journal.pone.0298895)
Supplement: S1 Table — (DOCX) [file pone.0298895.s004.docx]

**S1 Table 1. State variables of agricultural units**

| **Variables names** | **Meaning** | **Units** | **Status** |
| --- | --- | --- | --- |
| *Location* | Geographical location | X, Y | Static |
| *Distance-to-towns* | Distance to nearest city (Euclidian) | Kilometers | Static |
| *Production* | Annual harvest | Tons | Dynamic |
| *Price_production* | Harvest price | Sesterces | Dynamic |
| *Distance_cost* | Transportation cost | Sesterces | Dynamic |
| *Benefit* | Benefits | Sesterces | Dynamic |
