# Supplementary material for: The impact of climate change on the agriculture and the economy of Southern Gaul: New perspectives of agent-based modelling
Source: PLoS One. 2024 Mar 27;19(3):e0298895. doi: 10.1371/journal.pone.0298895 (PMC10971770; doi:10.1371/journal.pone.0298895)
Supplement: S2 Table — (DOCX) [file pone.0298895.s005.docx]

**S1 Table 2. State variables of grid cells**

| **Variables names** | **Meaning** | **Units** | **Status** |
| --- | --- | --- | --- |
| *Location* | Geographical location | X, Y | Static |
| *P_yield_wine* | Potential yield for wine | Hectoliters/hectare | Dynamic |
| *P_yield_oil* | Potential yield for olive oil | Hectoliters/hectare | Dynamic |
| *P_yield_cereals* | Potential yield for cereals | Tons/hectare | Dynamic |
| *Capitals_of_city?* | Presence/absence of a capital of city | Yes/no | Static |
