# Supplementary material for: The impact of climate change on the agriculture and the economy of Southern Gaul: New perspectives of agent-based modelling
Source: PLoS One. 2024 Mar 27;19(3):e0298895. doi: 10.1371/journal.pone.0298895 (PMC10971770; doi:10.1371/journal.pone.0298895)
Supplement: S3 Table — (DOCX) [file pone.0298895.s006.docx]

**S1 Table 3. Seasonal climate variables**

|  | **Variable name** | **Variable type and unit** | **Meaning** | **Calculation** |
| --- | --- | --- | --- | --- |
| 1 | *Thiv* | Floating point number;°C | Average winter temperatures | = (T12 + T1 +T2) /3 |
| 2 | *Tprt* |  | Average spring temperatures | = (T3 + T4 +T5) /3 |
| 3 | *Tete* |  | Average summer temperatures | = (T6+ T7 +T8) /3 |
| 4 | *Taut* |  | Average autumn temperatures | = (T9 + T10 +T11) /3 |
| 5 | *Phiv* | Floating point number; mm | Average winter precipitation | = (P12 + P1 +P2) /3 |
| 6 | *Pprt* |  | Average spring precipitation | = (P3 + P4 +P5) /3 |
| 7 | *Pete* |  | Average summer precipitation | = (P6+ P7 +P8) /3 |
| 8 | *Paut* |  | Average autumn precipitation | = (P9 + P10 +P11) /3 |
