# Supplementary material for: The impact of climate change on the agriculture and the economy of Southern Gaul: New perspectives of agent-based modelling
Source: PLoS One. 2024 Mar 27;19(3):e0298895. doi: 10.1371/journal.pone.0298895 (PMC10971770; doi:10.1371/journal.pone.0298895)
Supplement: S4 Table — (DOCX) [file pone.0298895.s007.docx]

**S1 Table 4. Prices given by the Edict of Diocletian (301 AD)**

| **Product** | **Name** | **Maximum price** |
| --- | --- | --- |
| Wine | *Vini rustici* | 8 denarii/sextarius |
|  | *Piceni*, *Tiburtini*, *Sabini* | 30 denarii/sextarius |
| Olive oil | *Olei Raphanini* | 8 denarii/sextarius |
|  | *Olei Floris* | 30 denarii/sextarius |
| Wheat |  | 100 denarii/modius |
| Transport costs |  | 20 denarii/roman mile/cart of 1200 pounds |
