# Supplementary material for: The impact of climate change on the agriculture and the economy of Southern Gaul: New perspectives of agent-based modelling
Source: PLoS One. 2024 Mar 27;19(3):e0298895. doi: 10.1371/journal.pone.0298895 (PMC10971770; doi:10.1371/journal.pone.0298895)
Supplement: S5 Table — (DOCX) [file pone.0298895.s008.docx]

**S2 Table 1. Minimum, average, and maximum values of each parameter used for the sensitivity analysis (SA)**

| **Factor** | **Unit** | **Minimum** | **Average (baseline)** | **Maximum** |
| --- | --- | --- | --- | --- |
| Temperature | Degree Celsius | -2° C | Climate averages used for each century in the model (here 1700 BP) | +2° C |
| Rainfall | mm | -100 mm |  | + 100 mm |
| Wine prices | *Denarii*/s*extarius* | 0 | 15 | 30 |
| Olive Oil prices | *Denarii*/s*extarius* | 0 | 20 | 40 |
| Cereal prices | *Denarii*/m*odius* | 0 | 50 | 100 |
| Transportation costs | *Sestertius*/kg/km | 0 | 0.07 | 0.14 |
