# Supplementary figures and images for: The impact of climate change on the agriculture and the economy of Southern Gaul: New perspectives of agent-based modelling
Source: PLoS One. 2024 Mar 27;19(3):e0298895. doi: 10.1371/journal.pone.0298895 (PMC10971770; doi:10.1371/journal.pone.0298895)

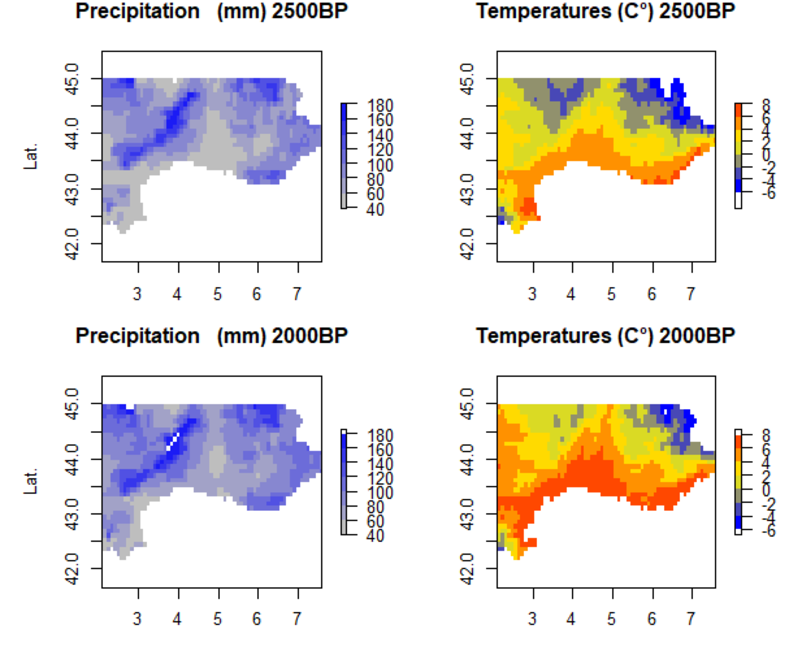

Supplement: S1 Fig — (TIF) [file pone.0298895.s009.tif]

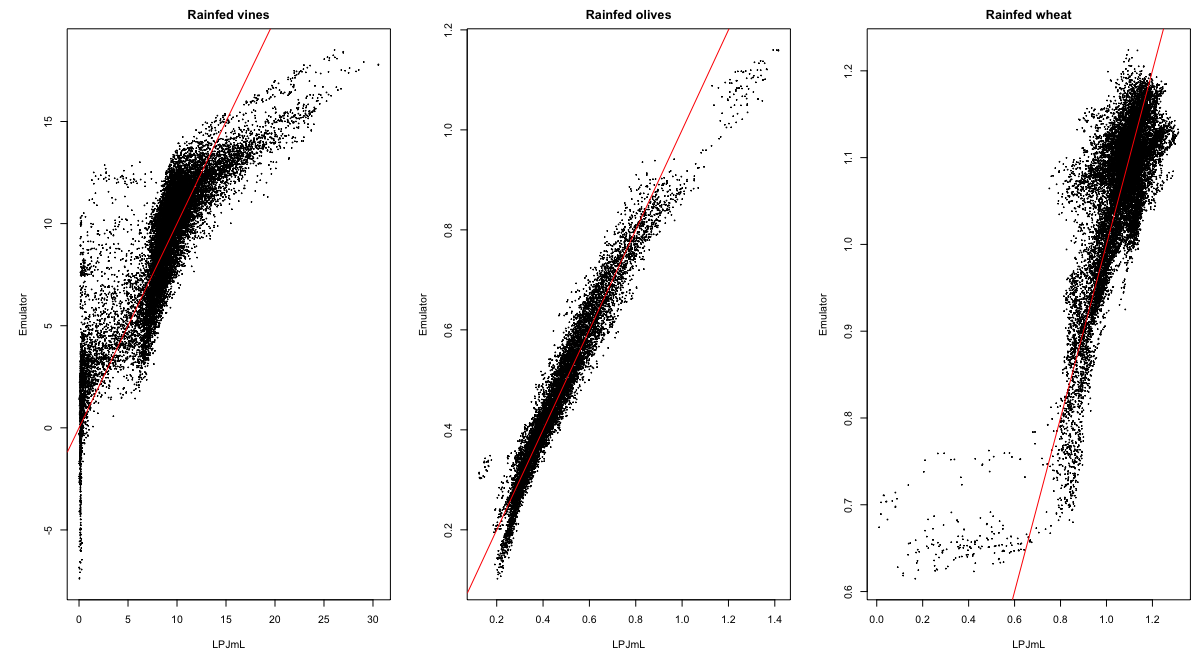

Supplement: S2 Fig — (TIF) [file pone.0298895.s010.tif]

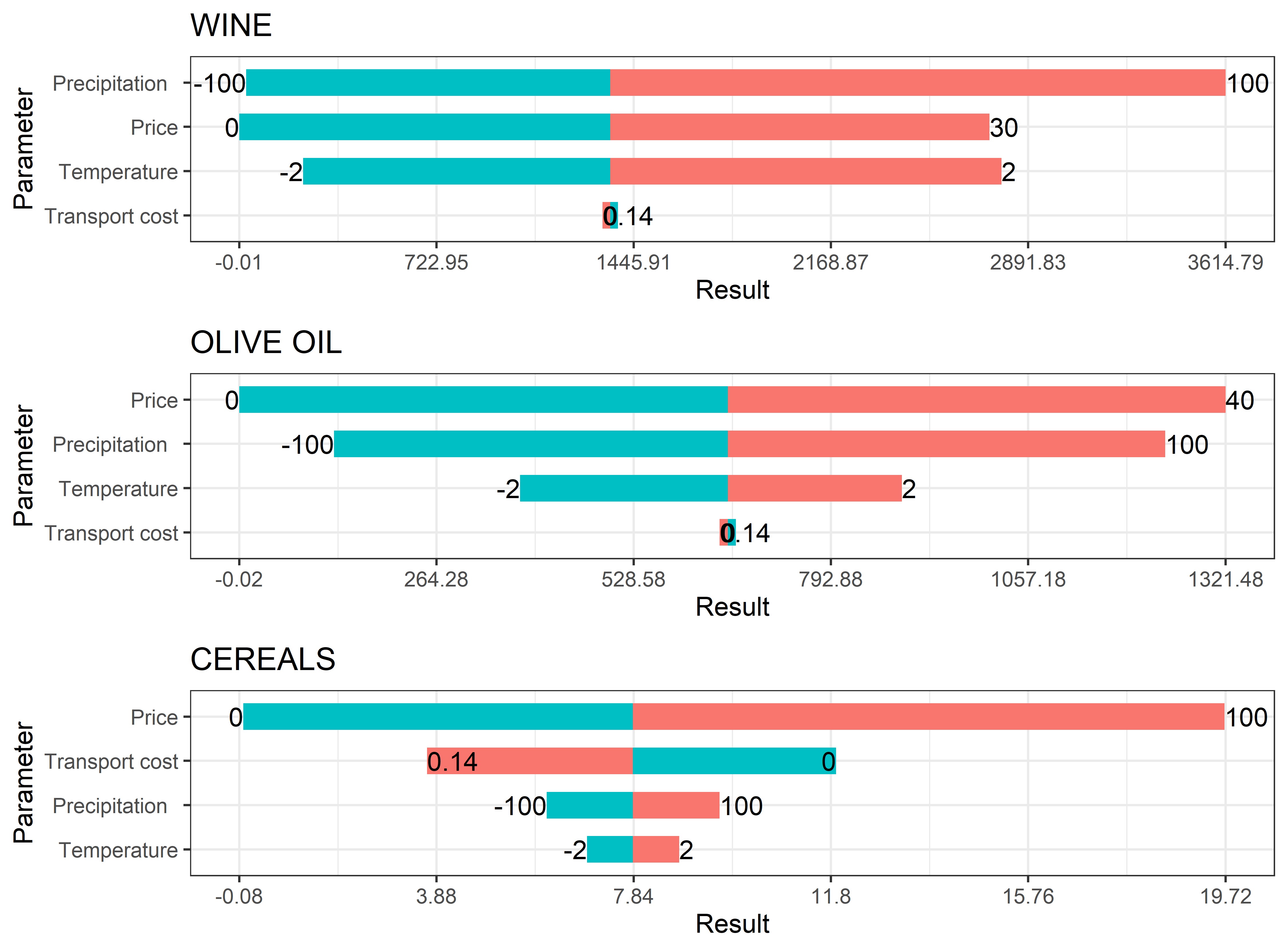

Supplement: S3 Fig — The parameters are ranked from top to bottom from most to least important. The data on the abscissa represent the sum of the benefits (in millions of sestertii) generated by all virtual agricultural exploitations. (TIF) [file pone.0298895.s011.tif]
