# Supplementary material for: The impact of climate change on the agriculture and the economy of Southern Gaul: New perspectives of agent-based modelling
Source: PLoS One. 2024 Mar 27;19(3):e0298895. doi: 10.1371/journal.pone.0298895 (PMC10971770; doi:10.1371/journal.pone.0298895)
Supplement: Attachment — Submitted filename: Response to Reviewers.docx [file pone.0298895.s013.docx]

**Review Comments to the Author**

**Reviewer #1**: The paper sets out to solve a clearly defined research problem through agent-based modelling, i.e. estimating the effects of climate change on the profitability of agrarian production in S. France before and during the Roman period. The paper is a good example of how a model with a relatively simple setup may yield important results that can be validated against the archaeological and historical record. The paper is clearly structured and describes the subject matter and results in clear terms, and provides a balanced discussion on how the results can inform archaeological and historical debate and research. To some extent, this goes at the expense of the detail on the modelling process itself, which is given rather scant attention. Issues of sensitivity analysis and comparison of scenarios are not discussed at all. This leads to a few questions that I would still like to see addressed in the paper:

[**1**] ABM is introduced without much background, and is arguably not justified as the best choice for this research problem. While I don't suggest to write a complete introduction to ABM, it will be good to apply the arguments for using ABM that have been made by e.g. Iza Romanowska and colleagues in their recent handbook: <https://www.santafe.edu/news-center/news/new-book-agent-based-modeling-archaeology>

[1]**->Authors answer**: indeed, the use of ABM in archaeology wasn’t really exposed and this was lacking. So, we have added in the main text a short new paragraph in which we make a brief state of the art about ABM and archaeology (lines 201-220). We cite here main references on the subject and the recent book of Romanowska, Wren and Crabtree (2021), which is a new milestone for this research. We explain too in this section why ABM is a promising tool for the study of the interactions between ancient societies and their environment, which are quite complex.

[**2**] the geographical resolution chosen is 8x8 km, because the climate data do not allow for a finer detail (or is also due to calculation limitations?); however, if only one exploitation of 25-60 ha is allowed per cell, then this cannot be considered very realistic – how was this tackled exactly in the model, and what consequences does this have?

[2]**->Authors answer**: for the model we decide to keep the original resolution of climate model (8x8 km, *i.e.* 64 km^2^). To work at a finer resolution, it would be necessary to downscale the climate data. We could have done this, even if this process is not an easy task. But our main problem was more here the calculation limitations with a desktop computer. Even with the “low” resolution of 8x8 km, the simulations launched with ROMCLIM are slow.

In ROMCLIM, one winery, one olive grove and one farm generated in a square of 64 km^2^ is equivalent to a density of 0.156 agricultural exploitation/km^2^ for each type (or 0.04 for the three). It seems low, but what was really the density of agricultural settlements in southern Gaul during Antiquity? This question remains quite difficult. We will take here the example of the Var department where the archaeological surveys were very intensive. Here, JP Brun mapped 103 olive groves (some of them could be wineries) for all the department (5973 km^2^), *i.e.* 0.01 olive grove/km^2^. Obviously, this ratio must be considered as a minimum. We perceive by archaeology only a part of the agricultural exploitations (real density is still very difficult to estimate). But we observe here that the density generated in ROMCLIM (0.125 olive grove/km^2^) is teen higher than the archaeological density observed in the Var. So surprisingly the density of exploitations generated in ROMCLIM can’t be considered as low, quite the contrary. To be clearer on this point, we have added in the main text these explanations (lines 364-381).

[**3**] not changing the prices is a major assumption, and the justification is only given in S2; some more discussion of this is warranted in the paper itself; in the end, it is not the prices as fixed numbers, but the factors influencing prices (technological, socio-political, economic) that should be the focus, maybe not of this paper, but certainly of future modelling and research

[3]**->Authors answer**: in the main text of the 1^st^ version, we present the results of one simulation for which we keep fixed prices and costs to better isolate the effects of climate change on agricultural revenues for each of the 13 centuries taken in account. Obviously, this was not realistic, because prices have changed during centuries. We have nevertheless improved our presentation of model outputs. Now, we present the potential effects of change in prices between a minimum and a maximum (lines 306-320). Prices still not vary in time, but the plots show all the possibilities on agricultural benefits with a realistic range of prices.

We want to precise that the observer can freely change the prices as he wanted. So, it’s possible to change them anytime according to some indications given by historical sources (or randomly). But we are totally agreeing that it will be more interesting to process theses prices by interactions between various factors, and not use them as input data. As suggested by the reviewer (and considering the difficulty of this specific issue) this could be a challenge for another agent-based model.

[**4**] the results are presented as if they are single outcomes – but with simulation modelling the number of runs determines how narrow or broad these outcomes are; can you provide more detail on this, perhaps in an appendix? The Github repository does not provide an extensive model (ODD or similar) description.

[4]**->Authors answer**: we are agreeing that the presentation wasn’t enough exhaustive concerning all the outputs possible. So, we have included in an appendix a Sensitivity Analysis (see S2), which reveals the sensitivity of the outputs to the four main variables of the model (temperatures, precipitation, prices, transport costs). The results show clearly how narrow or broad the outcomes are, as requested. Moreover, for a better overview of ROMCLIM, we have written in an appendix (S1) a complete standardized ODD protocol.

[**5**] Upon resubmission, please pay attention to text editing. While the English is generally of a good level, I noticed numerous small grammatical errors (in particular in plural / singular, as well as in present / paste tense) and some mistakes in the vocabulary. A few sentences were also a bit more convoluted than necessary. ->Ok, thanks, we have improved this.

Especially in the Discussion I found a number of terms that need to be revised, but please give the whole text a thorough check on consistency between English, French, Latin and Greek terms. Marseilles (English) vs. Marseille (French) ->Ok, thanks, corrections are done. L. 206: Histoire Universelle -> use Latin / Greek titles throughout, not French ones ->Ok, done. L. 209: emporions -> emporia ->Ok, done. L. 262: orthostats -> orthostates <https://en.wikipedia.org/wiki/Orthostates> ->Ok, done. L. 295: bare wheat -> naked wheat; clothed wheat -> hulled wheat; starch -> emmer; engrain -> einkorn ->Ok, done. L. 301: soft wheat -> common wheat / bread wheat (T. aestivum); durum wheat is either T. durum or T. turgidum ssp. Durum ->Ok, done. Also, the term 'juger' is not commonly used in scientific articles, preferred is 'iugerum' and its plural 'iugera'. ->Ok, done. In S1 and S2 I found the term 'olivettes' untranslated. ->Ok, done. 'Postulates' in S2 would be better termed 'assumptions'. ->Ok, done.

Remark: the corrections were done, but finally we decide to remove this paragraph from the text, as reviewer#2 asked to change the structure.

**Reviewer #2**: The approach and the model are interesting and worthy of note. The results presented so far seem promising. I truly do like the model, but the paper needs extensive revisions on every level.

[**1**] Research question / hypotheses: clearly defined research questions / hypotheses are lacking, the general question whether climate change influenced crop yield and the economy are too vague and can only serve as general framework. When reading the discussion it becomes clear that there are specific research questions, but they need to be made explicit and included in the introduction.

**[1]->Authors answer**: we are agreeing that the impact of climate change on crop yield and economy is a very generic question, but our model is truly built around it. Applied to our Study Area, we can specify that more specific question is to reconstruct a potential geography of agricultural production. Where was potentially the most rentable location to invest in oleiculture, or viticulture or cerealiculture? We have added some sentences in the introduction to make more clearer these more specific questions (see lines 54-60).

[**2**] The focus on „cash crops“ versus „food crops“ in general needs to be specifically stated and argued. Why is the consideration of cash crops more significant to your approach than „food crops“ in general. I am aware that part of this is formulated in the Supporting Information, but these are the foundations of your chosen approach and therefore need to be integrated in the main text.

[2]**->Authors answer**: this point wasn’t enough explained indeed. We focus to market-oriented agricultural production because they produce money. So, the impact of Climate Change on Roman economy (mainly funded on agriculture revenues) can be evaluated by these “cash crops”. Obviously, peasants cultivated beside various cultures for their own consumption, but these “food crops” were not sale. So, we don’t take them in account in ROMCLIM because it isn’t necessary for our purpose (and moreover very difficult to modelling because a huge lack of data and information on these cultures). It doesn’t mean that we consider that these “food crops” have only a little importance in agriculture. To better explain these considerations, we have added sentences in the main text (lines 167-179).

[**3**] State of the Art and presentation of study area: This is entirely lacking. The following topics need to be addressed (shortly): impact of climate variation on roman economy/agriculture, ABM and Archaeology, and ABMs addressing impact of climate variation on ancient civilizations.

[3]**->Authors answer**: we must admit that all this useful information was missing. So, we have added a new “State of the Art” section including all the topics mentioned: climate & roman economy, ABM & Archaeology, ABM & climate-ancient societies relationship (lines 83-220).

[**4**] Bibliography: Bernigaud et al. 2021, JAS Rep: I might have overlooked this, but there does not seem to be a reference to this previous work. The paper should be mentioned in the introduction. An explanation is required as to where new data comes in.

[4]**->Authors answer**: thank you, indeed we forget to mention this previous work dedicated to roman viticulture in Gaul, maybe because this wasn’t an ABM approach. But we added this reference called in the paragraph dedicated to the state of the art in viticulture (see line 108-109).

[**5**] Also, standard literature on ABM in Archaeoloy and Roman studies should be integrated such as Lake 2020, Mandich et al. 2019, Romanowska et al. 2021.

[5]**->Authors answer**: yes, it is useful for our purpose. Following the previous suggestion [3], we have added a little paragraph in the new “State of the Art” section about ABM & Archaeology with some lines on Roman studies, which are now more open to “formal models”. We have added here the references suggested by the reviewer (lines 202-221). However, we don’t find the reference Mandich et al. 2019, but we strongly assumed it might be Brughmans et al. 2019 (we are pretty sure that it is the same). Moreover, we have added a reference to the very recent book of Romanowska et al. 2021 (as previously suggested too by the reviewer#1).

[**6**] This paper presents a spatially explicit model! The research area needs to be presented: i) natural environment, ii) state of knowledge pertaining to land use (vine, olive and grain agriculture) in the study period. Large parts of the discussion can be used for this.

[6]**->Authors answer**: we perfectly agreeing this suggestion. So, we have added a short geographical presentation of the area (lines 66-80) and a brief state of the art on our knowledge about the “Mediterranean triad” (lines 86-148). Some elements were taken from the previous discussion section.

[**7**] Model description: Please use a standardized protocols such as ODD or Romanoswka 2021 for model description and include in the main text. The description in its present form is insufficient, it does not allow a good overview over the model and connected concepts.

[7]**->Authors answer**: yes, this was lacking indeed, as mentioned too by the reviewer#1. So, we have written an ODD protocol as an S1 (we have considered putting it in the main text, but it appeared to detailed).

[**8**] Model environment: Please explain the choice of the cell size: on pp. 4 cell size is argued with the resolution of the climate data, but on pp. 5 it is stated that the climate data used was at Mediterranean scale and was then interpolated to the 8 km scale.

[8]**->Authors answer**: this was unclear indeed. Climate reconstructions for Southern Gaul were processed with two sets of data. We have interpolated a low resolution (5’) paleoclimatic anomalies data at the finer resolution (8x8 km) of an actual modelling of the climate of France (ALADIN). The choice of keeping this resolution for the ABM is briefly specified in the main text (line 257-258). We don’t have chosen a finer, mainly for limitations in calculations with the ABM and a standard computer (on this point see my response to the reviewer#1, remark [2]).

[**9**] Presentation of Output data: Data is not presented adequately. Full output data must be included in the supplemental materials. As to data analysis, one graph and one map are not sufficient. A systematic overview over all analyzed data is necessary. What methods were used? Present the full output of your data analysis.

[9]**->Authors answer**: for a better presentation of all the outputs possible with this model, we have performed a Sensitivity Analysis (SA) which show the sensitivity of the “potential benefits” to the fourth main parameters (temperatures, precipitation, market price, transport costs). This SA performed with a One-factor-at-a-Time (OFAT) approach is now given in the S2 appendix.

[**10**] Discussion: The discussion needs to be reworked. Large parts of the discussion constitute state of the art material. In contrast a thorough discussion of the model output and new insight gained through the model is lacking.

[10]**->Authors answer**: main historical data previously presented in the discussion part are now replaced in the new section “State of the Art” (lines 83-221). In this discussion part, we have added a new paragraph in which we discuss more about the outputs and new insights (lines 365-424).
