# Supplementary material for: The impact of climate change on the agriculture and the economy of Southern Gaul: New perspectives of agent-based modelling
Source: PLoS One. 2024 Mar 27;19(3):e0298895. doi: 10.1371/journal.pone.0298895 (PMC10971770; doi:10.1371/journal.pone.0298895)
Supplement: Attachment — Submitted filename: Response to Reviewers.docx [file pone.0298895.s015.docx]

**Response to Reviewers:**

Reviewer's Responses to Questions

**Reviewer #2**: The authors have addressed most of my concerns, both in the responses and in the text. As I understand now, these were more due to how I understood the text, rather than to problems in the text itself. In any case, the clarifications included at the relevant parts of the text might help other readers to understand better the extent and objectives of the model.

On a particularly positive note, the historiography on ABMs applied to archaeology has greatly improved both in terms of content and references. At this stage, I would only have some small notes to add, for the authors to consider.

Line 28. Please, change ‘process’ by ‘processes’.

**Authors**-> Thanks, done.

Line 186. Please, change ‘agronomists’ by ‘agronomist’.

**Authors**-> The Saserna agronomists were both: so, the plural is accurate.

Line 187. Please, consider changing “gain to” by “gain for”.

**Authors**-> Thanks, done.

Line 305. Please, consider changing “suited to” by “suited for”.

**Authors**-> Thanks, done.

Line 310. Please, consider changing “doesn’t” by “does not”, more appropriate to the academic register. In general, please substitute all the abbreviations by their non-abbreviated counterpart when applicable.

**Authors**-> Thanks, done.

Line 354. Parallelisation and multicore computation can be done in any OS. Although I myself am a Linux user and supporter, why would Linux be a requirement? Perhaps it is not necessary to mention this at all in the text, but if the authors do want to reference it, perhaps refer more broadly to customary use on UNIX/Slurm on computer clusters?

**Authors**-> The developers of the LPJmL model work in a Linux environment because they estimate it’s the more practical choice (even if now other solutions in other OS are good). But we mention more explicitly SLURM in line 354.

7. PLOS authors have the option to publish the peer review history of their article ([what does this mean?](https://journals.plos.org/plosone/s/editorial-and-peer-review-process#loc-peer-review-history)). If published, this will include your full peer review and any attached files.

If you choose “no”, your identity will remain anonymous but your review may still be made public.

**Do you want your identity to be public for this peer review?** For information about this choice, including consent withdrawal, please see our [Privacy Policy](https://www.plos.org/privacy-policy).

Reviewer #2: No
